# Development of a Clostridium Perfringens Challenge Model in Broiler Chickens to Evaluate the Effects of Feed Additives

**DOI:** 10.3390/pathogens14070707

**Published:** 2025-07-17

**Authors:** Anna Kollár, Kinga Selymes, Gergely Tóth, Sándor Szekeres, Péter Ferenc Dobra, Krisztina Bárdos, László Ózsvári, Zsófia Bata, Viviána Molnár-Nagy, Miklós Tenk

**Affiliations:** 1 Department of Microbiology and Infectious Diseases, University of Veterinary Medicine, Hungária krt. 23-25, 1143 Budapest, Hungary; selyk55@gmail.com (K.S.); toth.gergely@univet.hu (G.T.); tenk.miklos@univet.hu (M.T.); 2 National Laboratory of Infectious Animal Diseases, Antimicrobial Resistance, Veterinary Public Health and Food Chain Safety, University of Veterinary Medicine, 1078 Budapest, Hungary; bardos.krisztina@univet.hu (K.B.); ozsvari.laszlo@univet.hu (L.Ó.); 3 Department of Parasitology and Zoology, University of Veterinary Medicine, István Street 2, 1078 Budapest, Hungary; szekeres.sandor@univet.hu; 4 HUN-REN-UVMB Climate Change: New Blood-Sucking Parasites and Vector-Borne Pathogens Research Group, 1078 Budapest, Hungary; 5 Department of Pathology, University of Veterinary Medicine, István Street 2, 1078 Budapest, Hungary; dobra.peter@univet.hu; 6 Department of Veterinary Forensics and Economics, Institute of Economics and Biostatistics, University of Veterinary Medicine, István Street 2, 1078 Budapest, Hungary; 7 Dr. Bata Ltd., 2364 Ócsa, Hungary; sbata@drbata.com (Z.B.); research@drbata.com (V.M.-N.)

**Keywords:** *Clostridium perfringens*, necrotic enteritis, challenge model, feed additives

## Abstract

Necrotic enteritis, caused by *Clostridium perfringens* (*C. perfringens*) is a disease present worldwide and causes major economic losses. The re-emergence of the disease, in recent years, is mainly due to the ban of the usage of antibiotics as growth promoters in the EU. The aim of this study was to establish a reliable, robust challenge model. Ross hybrid broilers were divided into randomized groups: a positive and a negative control group, a group receiving antibiotic treatment and three groups fed with assorted feed supplements, all receiving the same basal diet. The birds in the treatment groups were vaccinated twice using a 10-times dose of an Infectious Bursitis live vaccine and the animals were challenged four times with a NetB toxin producing *C. perfringens* strain. The presence of clinical signs and body weight gain were monitored. At the end of the study necropsy was performed and the gut lesions were scored. During the experiment, clinical signs were absent in the negative control group and in the antibiotic treated group. The other animals displayed diarrhea and feather loss. These symptoms were the most pronounced in the positive control group. The gut lesion scores showed significant differences between the negative and positive control groups, with the former scoring the lowest. Based on these results, the challenge model establishment was successful and in this setup the assessment of the potency of feed additives is also possible.

## 1. Introduction

Nowadays, as the population of the world is increasing rapidly, it is becoming more challenging to produce animal proteins in an efficacious and economic way. In the poultry industry, necrotic enteritis caused by *C. perfringens* is one of the diseases causing major economic losses [1]. Based on assessments, every year an approximately USD 6 billion loss is encountered worldwide, mainly due to the decreased body weight gain, higher feed conversion ratio and increased mortality [2]. Recently, the importance of the disease has been growing because of the ban of using antibiotics as growth promoters in the EU [1,3]. In the countries of the EU, both the clinical and subclinical forms of necrotic enteritis have started to occur more frequently [4].

Several factors influence the severity of the disease. In addition to the virulence factors of *C. perfringens*, secondary infections like coccidiosis, other immunosuppressive agents (e.g., infectious bursal disease), stress factors, feed and feed additives may change the outcome of necrotic enteritis [5].

*C. perfringens* is a Gram-positive, spore-forming rod, which is extremely resistant in its spore-forming form. It occurs in the soil and in the intestine of birds and mammalian species. The colonization with the bacteria starts at a very young age. As high as 75–95% of the broiler chickens carry *C. perfringens* [6]. The colony forming unit (CFU) number of *C. perfringens* in healthy animals is around 10^2^–10^4^ CFU/g, while in sick animals may reach 10^7^–10^9^ CFU/g [7]. The genetical analysis of *C. perfringens* proved that these bacteria do not have the key enzymes for amino acid synthesis; consequently, the essential substrates for their propagation are gained from the host with proteolytic and saccharolytic enzymes and specific transport systems [8]. In a limited amount of time these bacteria manage to overgrow the normal microbiome of the host intestine. Among the various toxins produced by the different *C. perfringens* strains, the NetB was identified as the most important one in evoking the disease [9,10]. As NetB is coded on a plasmid, it may be lost during the culture of bacteria in the laboratory, so it is advised to test the colonies for the presence of the toxin before the start of challenge trials [11].

The disease mainly occurs in 2–6-week-old broilers either in clinical or subclinical form [6,12]. The clinical form of necrotic enteritis is usually very rapid with high rate of mortality; the main clinical signs are apathy, ruffled feathers, loss of appetite, yellowish, foamy diarrhea and subsequent exsiccation [6]. In some cases, there is a rapid increase in the mortality rate (1% daily, 50% overall) without clinical signs [2,13]. In the case of the subclinical form, there is no significant increase in mortality, but the decreased body weight gain and the higher feed conversion ratio are indicative of the disease [7]. Although the latter form may be less visible, its economic impact is huge due to the higher prevalence and undetected background [1,2]. Gross pathological findings are characteristic: the wall of the small intestine is thinner, fragile, necrosis may be focal (mainly in the jejunum) but in more severe cases the ileum, duodenum or even the caecum is affected with large necrotic areas, covered with yellowish fibrin [2,12].

Necrotic enteritis is a typical multifactorial disease. The bacterium itself is wide-spread in poultry flocks but the clinical/subclinical form of the disease may be developed in the presence of other predisposing factors. The integrity of the intestinal wall is damaged, the mucous production is increased, there is a change in the composition of the microbiome, decrease in the transit time or damage of the immune system [6,12]. Around 3 weeks of age, when the maternal antibody levels of the birds start to decrease, the number of necrotic enteritis cases are increasing. Infections like Marek’s disease, infectious bursal disease, chicken infectious anemia and *Salmonella* Typhimurium were proven to promote necrotic enteritis [14]. Stress caused by overcrowding, temperature anomaly, drastic change of feed and the humidity of the litter are also key factors that are contributing to the overgrowth of *C. perfringens* in the gut [6,12,14].

The content and the physical properties of the feed are very important in the course of necrotic enteritis. The water-soluble non starch polysaccharides (NSPs) like beta glucans and methyl cellulose can be found in high amounts in rye, wheat, barley and oats, but the NSPs are not digestible for poultry [6]. The NSPs increase the viscosity of the intestine content and increase the transit time. As the enzymes of *C. perfringens* are able to cleave the NSPs, these serve as nutrients for the bacterial propagation and cause change in the composition of the microbiome [5,15]. Feeding poultry with NSP causes higher water intake, more diluted intestinal content and better conditions for the bacteria to propagate in the environment as well [12]. Several authors investigated these grains and observed 2–3-times higher mortality compared to a corn-based diet [16,17]. The severity of necrotic enteritis is in correlation with the protein content of the feed, especially in the case of proteins of fish origin [6,13] due to the high glycine and methionine content, which helps the propagation of *C. perfringens*.

In the broiler industry, antibiotic growth promoters are banned in the EU [18]. In order to control the colonization of the bacteria, the local intestinal microenvironment can be influenced with different feed additives like pro-, pre- and postbiotics, plant extracts, essential oils, organic acids or bacterial derived molecules [19]. Probiotics are live feed additives that help in creating and maintaining the microbiome balance. In the poultry industry the most commonly used probiotics are *Bacillus*, *Lactobacillus*, *Enterococcus* and *Bifidobacterium* species and *Saccharomyces* yeast [20,21]. Prebiotics contain non-digestible oligosaccharides like mannan-oligosaccharide in yeast, fructo-oligosaccharide in plants or inulin. Prebiotics may be applied alone or in combination with probiotics [19]; the latter are called synbiotics. Phytobiotics are plant-derived, biologically active feed additives that support directly the intestinal microbiome, inhibit the growth of pathogenic microorganisms or have a direct effect on the microenvironment in the intestine by changing its composition or pH [12]. In most cases different herbaceous plants are used for this purpose like curcuma, oregano, thyme, ginger, anise and clove [6]. Trace elements are also very important ingredients. Zinc is crucial in cell growth, in the development and proper functioning of the immune system and against oxidative stress. Manganese helps to maintain the integrity of the intestinal wall; copper, in high concentrations, may have a direct antimicrobial effect [22].

The establishment of a necrotic enteritis challenge model is very difficult, since it is a multifactorial disease. In the case of testing anti-clostridial drugs, the aim is to evoke the clinical form of necrotic enteritis. On the other hand, in those cases in which the prevention of necrotic enteritis should be examined, the subclinical form of the disease is tested with thorough investigation of body weight gain and feed conversion rate data [23]. The change of feed right before the *C. perfringens* challenge to another one with a high amount of animal-derived proteins and NSPs may also contribute to a more effective challenge model.

Several experiments were carried out for the investigation of necrotic enteritis; however, the complex pathomechanism of the disease and the high number of other influencing factors have not made it possible to create a reliable and reproducible challenge model yet [5].

The aims of our study were to establish a *C. perfringens* challenge model combining the available literature data (I) and to determine the suitability of the model for testing different feed additives (II).

## 2. Materials and Methods

### 2.1. Animals and Their Keeping Conditions

The animal experiment was conducted in accordance with the guidelines of Hungarian Government Decree No. 40/2013 (II.14.) and approved by the Ethical Committee of the Veterinary University of Budapest. This research was approved by the Pest County Government Office, Hungary in 2023 (license number: PE/EA/01317-6/2023). The severity of the animal experiment was categorized as moderate.

Altogether 129 day-old Ross 308 broiler chickens (males and females, mixed as they randomly arrived from the hatchery) were involved in the experiment, as the minimum statistically required number. The birds were vaccinated against Newcastle Disease and Infectious Bronchitis at the hatchery. After arrival, the animals were randomly allocated into six groups as they came to hand. Each chicken was labelled with leg-rings that were adjusted to the required size as the birds grew. The animals were kept in separated pens at the conventional animal house of Veterinary Medicine University Budapest, Department of Microbiology and Infectious Diseases (area: 3–4 square meters). The chickens received feed and fresh water ad libitum. The optimal temperature and lighting regimens of the breed were maintained throughout the experiment. The environment was enriched for the animals (e.g., with a sand bath). The experiment was blinded, the composition of the feed was not known by the investigators of the experiment and each feed was color-coded.

### 2.2. Feed

The groups received different diets; the compositions of the feeds are summarized in Table 1. The negative (Group 6) and positive control groups (Group 1), including the one receiving antibiotics (amoxicillin) after challenge (Group 5), received the same basal diet containing wheat, soybean meal, corn, sunflower oil and broiler premix without coccidiostats throughout the entire experiment. The antibiotic treatment was carried out in drinking water, the dose of amoxicillin was 13.1 mg/kg and the application was carried out according to the instructions of use provided by the manufacturer. The other three groups were fed with the basal diet supplemented with different prototype feed additives. One group (Group 2) received feed additive containing copper chelate, hop extract and chicory root. Another group (Group 3) received *Bacillus licheniformis*, corn cob and soybean meal. In Group 4 fenugreek extract, copper chelate, zinc chelate, chicory root and curcuma extract were used. In all groups (except the negative control group) the basal diet was replaced by a diet containing fish meal on day 17, before the challenge, to generate a protein rich environment in the gut. The feed was produced and provided by Dr. Bata Ltd.

### 2.3. Challenge Material

The *C. perfringens* strain (ID. No. 553/23) was received from the Department of Pharmacology, Veterinary Medicine of Budapest. The strain was originally isolated from a hen. Before challenge, the strain was tested for the presence of NetB gene with Kylt^®^ Clostridium perfringens PCR Kit (cat. no. 31034). The kit was used according to the user’s manual of the manufacturer. Briefly, the DNA was isolated with 5% Chelex^®^100 Resin solution (BioRad, Hercules, CA, USA, cat. no. 142-1253), then 4 µL of the solution was added to the PCR buffer mix. The PCR profile was as follows: denaturation at 95 °C for 10 min, then 42 cycles: 15 s at 95 °C, 1 min at 60 °C with fluorescent detection. The challenge strain was positive for NetB toxin. The bacteria were propagated in semifluid Thioglycolate PH EUR-USP medium (Biolab, Hungary, catalog number: THM40010) overnight, then the fresh culture was used for the challenge.

### 2.4. Animal Experiments and Summary of the Operations

For the summary of the operations during the experiment see Table 2. The animals were weighed upon arrival and six different groups containing either 21 or 22 birds were created as they came to hand; individual labelling was also performed. The body weight data were analyzed with ANOVA, using Statgraphics Centurion XVIII software; on day 0 no statistical difference (*p* < 0.05) was found between the groups. The chickens were monitored daily, clinical signs were recorded on the clinical observation data capture forms, especially the change in the consistency of the feces (bloody feces, contamination of the cloaca with feces, urate crystals), lameness, droopy wings, lethargy, change in behavior. Individual body weight measurement was carried out on days 0, 7, 18 and 25.

During the experiment the animals in Groups 1–5 were vaccinated twice with 10 times overdose of the Cevac Gumbo L vaccine. The vaccine contains an attenuated strain of infectious bursal disease virus propagated in cell culture, in freeze-dried form. The vaccine was applied individually with pipetting 0.2 mL vaccine orally on D14, then the vaccination was repeated on D21. On D17 the feed was partially changed: the soybean meal was replaced with high protein containing fish meal (except in the negative control group). The birds were challenged with 200 µL *C. perfringens* orally with a pipette (10^8^ CFU/animal). The animals were challenged on four consecutive days starting from D18 until D21. Fresh bacterial cultures were used for each inoculation. On D25 the animals were transported to the Pathology Department of Veterinary Medicine of Budapest and after euthanasia gross pathological examination and scoring were carried out. The trial was shorter than the average rearing period of the broilers since in these type of challenge models the characteristic gross pathological findings can be better observed at this age.

### 2.5. Evaluation of the Study

During the experiment clinical signs and deaths were recorded daily. The body weights of the animals were individually measured. Individual measurement of the consumption of the feed was not possible due to the deep litter applied in the study. At the end of the experiment the small intestine of the birds was observed and scored (0–6) based on the evaluation described previously [5], see Table 3. Lesion scoring was performed by one skilled pathology expert, for whom the treatment of the different groups was not known. The person involved in the evaluation of the gross pathological scores only recorded the serial number of the birds (without knowing the group) and the scores, then handed the results to those colleagues who collected the results in the raw data package.

### 2.6. Statistical Analysis

The statistical analysis was performed using Statgraphics Centurion version XVIII (The Plains, VA, USA). In the software, one-way analysis of variance (ANOVA) function was used. The grouped data was assessed by the factors, i.e., the body weights, body weight gain and the pathological changes. As limit of the statistically significant difference, the *p* value < 0.05 was used. The group means were compared using the multiple range tests (Tukey’s HSD method).

## 3. Results

### 3.1. Clinical Observation

During the experiment 12 animals died, 11 birds at the beginning of the study between days 4 and 8. After challenge in Groups 1–4 all animals had stinky, watery diarrhea until the end of the study and the skin surrounding the cloaca was featherless, especially in Group 1. There were no changes in the behavior of the birds. The list of birds found dead is summarized in Table 4.

### 3.2. Body Weight Data

**Figure 1:**
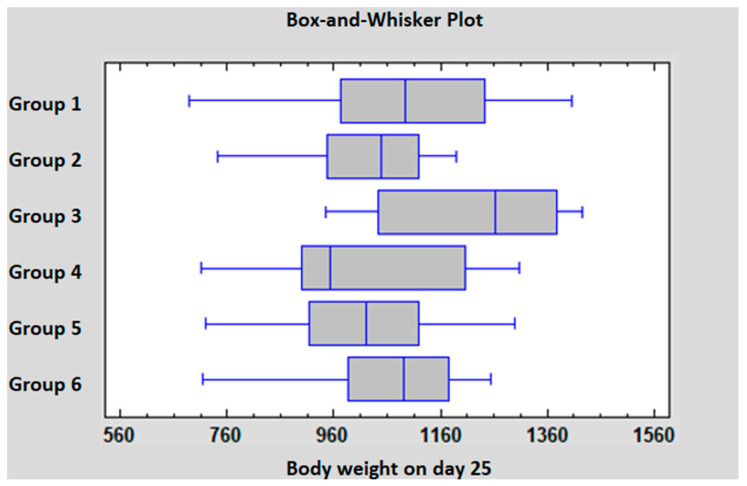
Statistical comparison of the body weight data on D25.

The individual body weight data are shown in Tables S1–S6. The group average body weight data are summarized in Table 5. Based on the statistical analysis on D18, there was a significant difference between the body weight of Groups 1, 3 and 6 compared to Groups 4 and 5. On D25 the body weight of Groups 3 and 6 was significantly higher compared to Group 2. The statistical analysis of D25 data is presented in Figure 1.

**Figure 2:**
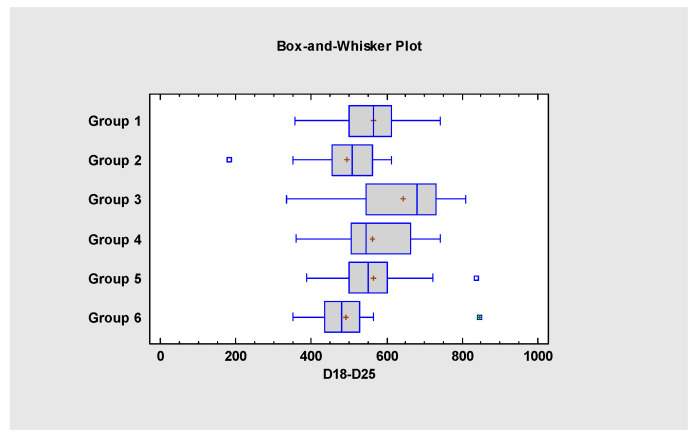
Statistical comparison of the body weight gain data from D18 to D25.

The average body weight gain is summarized in Table 6. Based on the statistical analysis, between D18 and D25, the body weight gain of Group 3 was significantly higher compared to all the other groups. In Groups 1, 4 and 5 the body weight gain was significantly higher compared to Groups 2 and 6. The statistical analysis of D18–D25 body weight gain data is presented in Figure 2.

### 3.3. Gross Pathology

**Figure 3:**
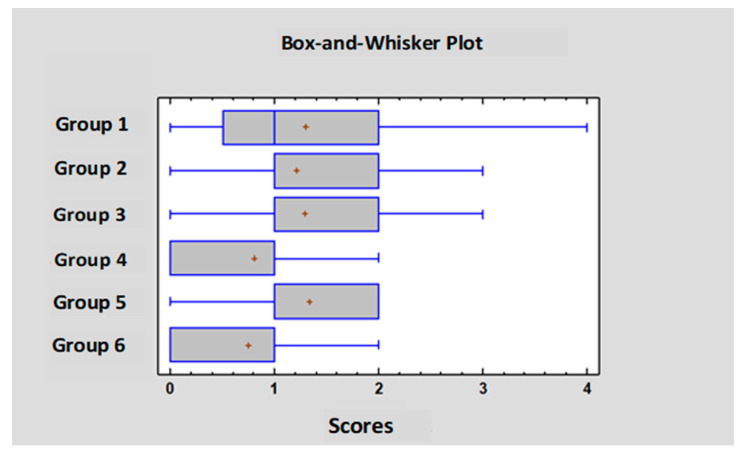
Statistical comparison of the gross pathology scores.

**Figure 4:**
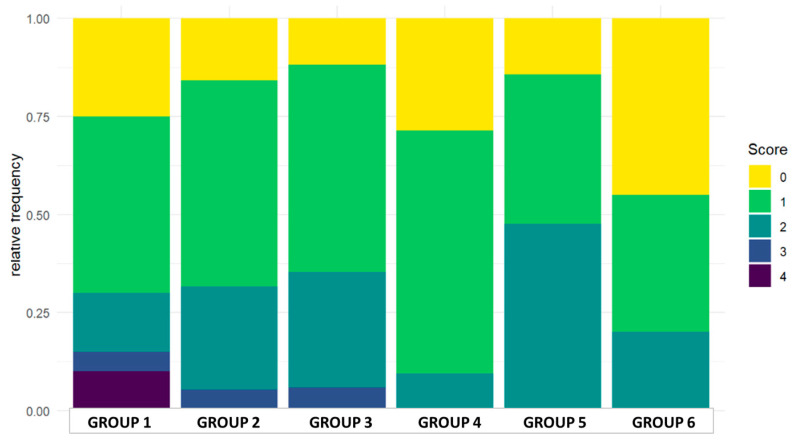
Distribution of the scores in the groups.

**Figure 5:**
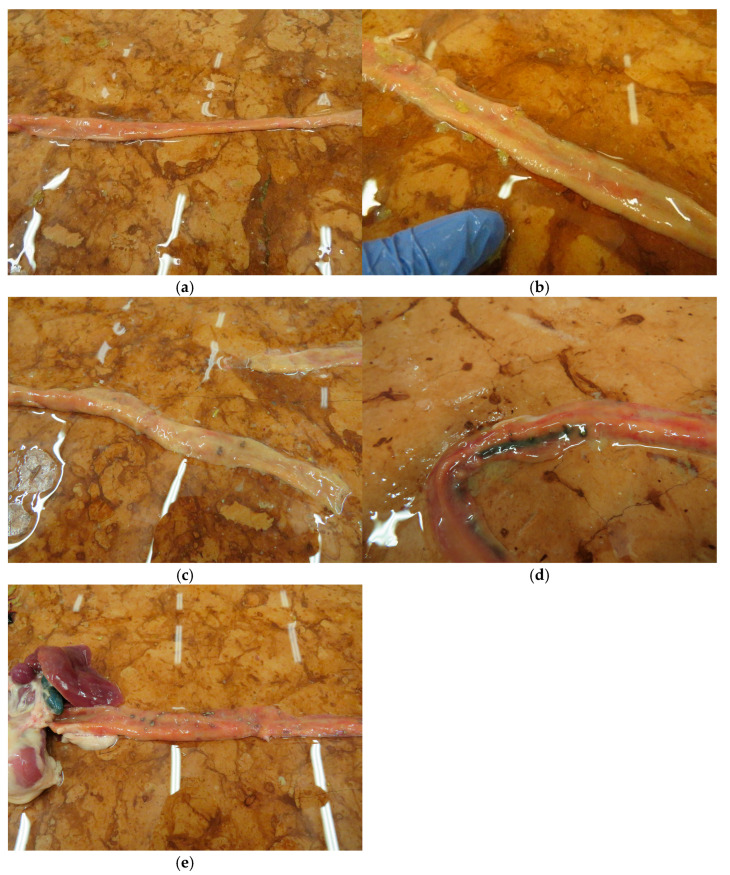
Representative small intestinal scores (**a**) score 0, (**b**) score 1, (**c**) score 2, (**d**) score 3, (**e**) score 4.

On day 25 the small intestine of the chickens was evaluated according to the scoring system described in Table 3. Scores between the range of 0 and 4 were found in the experiment; more severe pathological findings (scores 5 or 6) were not observed. Individual and group average data are shown in Tables S7, S8 and Table 7. Statistical analysis of the scores is shown in Figure 3. The occurrence of the different scores is summarized in Figure 4. Representative photos of the different scores are shown in Figure 5. The scores in Group 6 were significantly lower than those of Groups 1 and 5. In addition, in Group 4 the scores were significantly lower compared to Group 5.

## 4. Discussion

The experimental evocation of necrotic enteritis caused by *C. perfringens* is challenging, as the disease is multifactorial. The clinical and subclinical manifestation of the disease depends mainly on external circumstances and predisposing factors. In our study, the goal was to elaborate a reproducible method, in which the clinical and gross pathological lesions can be evoked for the closer observation of the disease in a controlled experiment. A *C. perfringens* strain with NetB toxin from a fresh bacterial culture is a key (15–24-h long incubation) for a successful experimental setup [5]. We applied an individual per os route challenge of the birds with 10^8^ CFU/mL freshly prepared bacterial solution for four consecutive days. Challenging with fresh bacterial cultures prepared with shorter incubation time has the advantage of creating more severe lesions compared to the older cultures [24]. Although the individual application of the challenge material is time-consuming, this way the close clinical observation of the animals is possible, including body weight measurement. In several previously described studies the challenge material was given in the feed or in the drinking water [25]; in these cases, the dose of the challenge bacteria may significantly differ in each bird. Feed and drinking water withdrawal may increase the uptake of the challenge material but raises animal welfare issues. A further disadvantage of the method is the higher amount of challenge material needed. An advantage of the technique is that the individual handling of the birds can be avoided.

As the application of a virulent *C. perfringens* strain alone does not cause necrotic enteritis in all of the cases, there is also a need for further predisposing elements, like pre-infection with coccidia (*E. maxima, E. acervulina, E. necatrix*) 4–5 days before the *C. perfringens* challenge with 2–5 × 10^4^ oocysts, or 10 × dose of a vaccine strain [5]. In these types of challenge experiments the feed composition does not influence notably the outcome of the disease, since the intestinal wall has already been damaged by the coccidia and the protein and amino acid supply of *C. perfringens* is appropriate [26]. As a precursor, infectious bursal disease vaccines with intermediate or intermediate plus strains in 10 times dose can also be applied 4–5 days before the *C. perfringens* challenge, then repeated after 7 days [27]. In our study we chose this method. The birds were vaccinated twice with a 10-times overdose of a registered live IBD vaccine. In our experiment the most severe clinical signs were observed in the positive control group. On the other hand, in the negative control group there was no diarrhea or loss of feathers. In the group treated with antibiotics, the medical treatment successfully prevented the appearance of the clinical signs. These findings are in agreement with previous studies [28,29]. The challenge itself did not cause any significant increase in mortality as only one animal died after the challenge. In Group 3 (fed with *Bacillus licheniformis* feed additive) the number of dead animals was high at the beginning of the experiment, while in Group 4 (fed with fenugreek copper and zinc chelate feed additive) all the animals were alive until the last day of the trial.

Based on the gross pathological findings, the experiment was successful as the scores in the positive control group were significantly higher than those of the negative control group in which the lowest scores were recorded. The highest score in the experiment was 4; all of these occurred in the positive control group. Scores in the range of 0–2 were observed in all the other groups too, including the negative control birds. In the negative control group, in the group treated with antibiotics and in the fenugreek, copper and zinc additive fed chickens the highest score was 2. The average score of the latter group was almost as low as that of the negative control. In the antibiotic-treated group, despite the lack of clinical signs, the average pathological scores were even higher than in the positive control. This finding may be due to the antibiotic’s harmful effect on the normal microbiome (I), or due to its direct damage to the intestinal mucosa (II). For the confirmation of this phenomenon, further laboratory investigations are needed. Unfortunately, there is no universal scoring system for the evaluation of the small intestinal lesions, several other scales were described, within a 0–3 or 0–4 range, which makes the comparison of the conducted trials difficult [5,25]. In general, similar to our experiment, the gross pathological scores correlated well with the appearance of the clinical signs. On the contrary, the body weight data in our experiment were ambiguous. On D25 only the average body weight of Group 3 was higher compared to the other groups. As in this group the number of dead chickens was the highest at the beginning of the experiment; the lower number of birds per square meter in the second half of the experiment may have caused this phenomenon. Importantly, the length of the experiment was shorter than the regular rearing period of broilers. Although in the negative control group the body weight gain was not above the average, this group was the most homogeneous with low standard deviation. On the other hand, the highest standard deviation was observed in the case of the positive control group. The negative control group received the same basal diet throughout the entire experiment, while from D17 all the other groups received a higher protein containing feed. Although the aim of the feed change was to implement it as a stressor and to help the effective challenge with *C. perfringens* (based on previous summarizing literature data [5]), our results suggest that the higher protein content may contributed later on to the increased body weight gain in the treatment groups. As the body weight gain data were not in agreement with those expected [11], in future trials a more homogeneous allocation of the animals will be needed and the close surveillance of the different parameters like density, light program, temperature. As it was already highlighted, in deep litter keeping conditions, the individual measurement of the feed intake is not feasible. The extension of the experiment until the end of the rearing period of the broilers (day 35–42) can also be an option for the better analysis of the results.

Among the tested feed additives, the fenugreek extract seemed to be promising based on the gross pathological findings. In other studies, this feed additive was also performing well against a Salmonella challenge [30,31]. Although the pro- and synbiotic feed additives seem to be promising antibiotic growth promoter alternatives by increasing the body weight gain and improving the feed conversion ratio [32,33], the potential resistance gene spreading has to be handled with caution [34]. Previously, the trace elements like zinc, manganese and copper were applied in non-organic form in the feed additives in very high concentrations, but recently these have been used as organic compounds, as chelates. The effects of these trace elements were proven in coccidiosis, but their mechanisms were not tested in detail in necrotic enteritis cases [22,35].

## 5. Conclusions

All the above confirms that the experimental model establishment of necrotic enteritis is still a challenging and complex task. Although our results were not congruent from all aspects, we consider our model based on the gross pathological scores successful, mainly due to the fact that fewer variables are behind the pathological scores and it is closely related with the infection itself, and the scores were in correlation with the clinical signs. The implementation of antibiotic free strategies in the poultry industry to combat against the disease will have an utmost importance in the future. As a key element of this new strategy, different feed additives may be tested prior to use with the help of this model.

## Figures and Tables

**Table 1:** Components of the basal diet during the experiment.

Basal Diet	%	Days	Form
Soybean meal *	35.6	0–25 days	Crumbled
Wheat	30.0		
Corn	27.4		
Sunflower oil	3.0		
Broiler premix without coccidiostats	4.0		

* From day 17 until day 25 soybean meal was changed to fish meal (except negative control).

**Table 2:** Summary of the operations.

SUMMARY OF THE OPERATIONS						
Groups/ Operations	**1**	**2**	3	4	5	6
Feed/ Number of Birds in the Group	Basal Diet Positive Control 21	Basal Diet + Feed Additive 21	Basal Diet + Feed Additive 22	Basal Diet + Feed Additive 21	Basal Diet + Amoxicillin 22	Basal Diet Negative Control 22
D0	Hatching, transport of day-old broilers to the Dept. of Microbiology and Infectious Diseases, University of Veterinary Medicine, Budapest Body weight measurement, individual labelling, group forming					
D7	Body weight measurement					
D14	Vaccination with IBD					-
D17	Feed change (fish meal, high protein content)					-
D18	Body weight measurement Challenge with *Clostridium perfringen*					Body weight measurement
D19	Challenge with *Clostridium perfringens*					-
D20	Challenge with *Clostridium perfringens*					-
D21	Challenge with *Clostridium perfringens* Vaccination with IBD					-
D25	Body weight measurement Transport of the birds to the Department of Pathology, University of Veterinary Medicine Budapest, euthanasia, gross pathology					

**Table 3:** Gross pathology scoring system.

Score	Description
0	No gross lesions
1	Thin/friable walls or diffuse superficial but removable fibrin
2	Focal necrosis/ulceration, non-removable fibrin deposit, 1–5 lesions
3	Focal necrosis/ulceration, non-removable fibrin deposit, 6–15 lesions
4	Focal necrosis/ulceration, non-removable fibrin deposit, 15 or more lesions
5	Patches of necrosis 2 to 3 cm long, variable number of lesions
6	Diffuse necrosis, variable number of lesions, extensive

**Table 4:** Data of dead animals during the experiment.

Serial Number	Day of the Experiment	Group Number	Identification Number of the Birds
1	D4	2	35
2		2	39
3		3	41
4		3	47
5		3	50
6		6	8
7		6	17
8	D5	1	1
9		3	45
10		3	46
11	D8	5	99
12	D24	3	42

**Table 5:** Group average body weight data based on the individual body weighing (mean ± SD).

Groups	D0 (g)	D7 (g)	D18 (g)	D25 (g)
G1 positive control (basal diet)	47.14 ± 3.2	127.95 ± 30.06	529.00 ± 99.56 ^a^	1092.55 ± 188.14
G2 basal diet + feed additive	45.9 ± 2.53	144 ± 36.18	503.21 ± 76.88	996.89 ± 162.99 ^b^
G3 basal diet + feed additive	46.91 ± 4.32	166 ± 31.34	567.47 ± 83.5 ^a^	1212.38 ± 181.06 ^a^
G4 basal diet + feed additive	48.14 ± 3.12	119.62 ± 29.02	464.86 ± 84.08 ^b^	1027.91 ± 182.34
G5 basal diet + amoxicillin	47.41 ± 3.45	151.32 ± 30.02	448.95 ± 75.19 ^b^	1014.19 ± 167.52
G6 negative control (basal diet)	47.18 ± 3.2	154.65 ± 33.17	568.95 ± 103.07 ^a^	1059.95 ± 154.6 ^a^

^a,b^ denote a significant difference.

**Table 6:** Average body weight gain data during the different phases of the experiment (mean ± SD).

Groups	Between D0 and D7 (g)	Between D7 and D18 (g)	Between D18 and D25 (g)
G1 positive control (basal diet)	80.81 ± 30.38	401.05 ± 74.94	563.55 ± 104.22 ^b^
G2 basal diet + feed additive	98.10 ± 36.07	359.21 ± 45.88	493.68 ± 101.08 ^a^
G3 basal diet + feed additive	119.09 ± 30.96	401.47 ± 59.10	644.90 ± 126.04 ^c^
G4 basal diet + feed additive	71.48 ± 29.56	345.24 ± 58.88	563.05 ± 110.81 ^b^
G5 basal diet + amoxicillin	103.91 ± 28.85	297.63 ± 49.49	565.24 ± 107.93 ^b^
G6 negative control (basal diet)	107.47 ± 33.02	414.30 ± 75.08	491.00 ± 102.92 ^a^
Mean	96.81	369.82	553.57

^a,b,c^ denote a significant difference.

**Table 7:** Group average gross pathology scores (mean ± SD).

Groups	Scores
G1 positive control (basal diet)	1.3 ± 1.22 ^b,c^
G2 basal diet + feed additive	1.21 ± 0.79
G3 basal diet + feed additive	1.29 ± 0.77
G4 basal diet + feed additive	0.81 ± 0.60 ^a,b^
G5 basal diet + amoxicillin	1.33 ± 0.73 ^c^
G6 negative control (basal diet)	0.75 ± 0.79 ^a^

^a,b,c^ denote a significant difference.

## Data Availability

The data presented in this study are in the manuscript.

## Supplementary Materials

The following supporting information can be downloaded at: https://www.mdpi.com/article/doi/s1.Table S1: Group 1 individual body weight data; Table S2: Group 2 individual body weight data; Table S3: Group 3 individual body weight data; Table S4: Group 4 individual body weight data; Table S5: Group 5 individual body weight data; Table S6: Group 6 individual body weight data; Table S7: Individual gross pathology scores in Groups 1, 2 and 3; Table S8: Individual gross pathology scores in Groups 4, 5 and 6.
