# Supplementary material for: Development of a Clostridium Perfringens Challenge Model in Broiler Chickens to Evaluate the Effects of Feed Additives
Source: Pathogens. 2025 Jul 17;14(7):707. doi: 10.3390/pathogens14070707 (PMC12300089; doi:10.3390/pathogens14070707)
Supplement: Supplementary file 1 [file pathogens-14-00707-s001.zip › pathogens-3724298-supplementary.pdf]

Table S1. Group 1 individual body weight data

Group 1 Positive control (basal diet)	Days			
**ID**	**D0**	**D7**	**D18**	**D25**
1	47	[redacted]		
2	45	81	414	906
3	42	128	603	1320
4	52	141	494	850
5	46	80	443	1053
6	43	147	583	1188
7	46	169	653	1247
8	43	154	510	1014
9	51	130	500	1052
10	45	170	660	1304
11	48	159	662	1405
12	49	86	322	688
13	50	165	648	1190
14	45	131	569	1303
15	52	122	437	931
16	45	125	496	1025
17	52	120	538	1117
18	48	114	526	1070
19	45	147	628	1241
20	45	72	360	827
101	51	118	534	1120
Mean	47.14	127.95	529.00	1092.55
SD	3.20	30.06	99.55	188.14

Table S2.. Group 2 individual body weight data

Group 2<br>Basal diet + feed additive	Days			
ID	D0	D7	D18	D25
21	43	122	504	1083
22	47	81	406	889
23	43	152	505	1011
24	44	160	498	947
25	48	181	548	1057
26	46	192	582	1122
27	44	166	577	1189
28	46	163	530	1040
29	49	158	540	1131
30	48	82	392	742
31	45	153	543	1106
32	46	70	331	744
33	52	179	612	1097
34	41	154	480	953
35	46	[redacted]		
36	46	164	528	1136
37	45	162	600	1120
38	44	143	498	1050
39	46	[redacted]		
40	50	159	507	962
102	45	95	380	562
Mean	45.90	144.00	503.21	996.89
SD	2.53	36.18	76.88	162.99

Table S3. Group 3 individual body weight data

Group 3 Basal diet + feed additive	Days			
**ID**	**D0**	**D7**	**D18**	**D25**
41	43	[redacted]	[redacted]	[redacted]
42	53	172	562	[redacted]
43	53	112	449	1073
44	40	125	502	1062
45	45	[redacted]	[redacted]	[redacted]
46	46	205	656	990
47	48	[redacted]	[redacted]	[redacted]
48	54	219	614	1335
49	47	203	656	1371
50	45	[redacted]	[redacted]	[redacted]
51	49	148	584	1394
52	45	134	427	954
53	47	178	655	1381
54	44	180	643	1425
55	42	151	538	1136
56	39	[redacted]	[redacted]	[redacted]
57	52	178	579	1223
58	45	157	492	1024
59	51	176	621	1374
60	53	129	421	944
103	44	150	576	1302
Fek23	47	205	672	1410
Mean	46.91	166.00	567.47	1212.38
SD	4.32	31.34	83.51	181.06

Table S4. Group 4 individual body weight data

Group 4<br>Basal diet + feed additive	Days			
**ID**	**D0**	**D7**	**D18**	**D25**
61	50	147	545	1207
62	43	123	507	1232
63	51	160	559	1232
64	52	92	407	912
65	53	143	569	1308
66	43	108	350	710
67	50	85	382	898
68	48	103	403	849
69	50	148	555	1191
70	53	95	380	934
71	49	155	602	1301
72	44	156	559	1300
73	47	87	406	903
74	44	106	385	893
75	50	65	357	901
76	47	149	582	1156
77	46	139	471	857
78	50	85	394	899
79	47	144	478	988
80	49	103	412	963
104	45	119	459	952
Mean	48.14	119.62	464.86	1027.90
SD	3.12	29.02	84.08	182.34

Table S5. Group 5 individual body weight data

Group 5 Basal diet + Amoxicillin	Days			
**ID**	**D0**	**D7**	**D18**	**D25**
81	46	165	467	1132
82	48	152	425	975
83	50	117	351	795
84	47	160	536	1120
85	51	177	511	1035
86	49	175	508	1182
87	44	68	296	720
88	47	157	417	864
89	53	166	425	924
90	51	175	523	1096
91	50	165	448	1022
92	48	167	463	1300
93	42	138	420	914
94	43	81	321	847
95	50	132	362	750
96	43	150	447	1048
97	44	144	431	958
98	44	151	453	1050
99	48	161	[redacted]	[redacted]
100	42	188	568	1290
105	51	156	477	1011
Fek24	52	184	579	1265
Mean	47.41	151.32	448.95	1014.19
SD	3.45	30.02	75.19	167.52

Table S6. Group 6 individual body weight data

**Group 6 Negative control Basal diet**	**Days**			
**ID**	**D0**	**D7**	**D18**	**D25**
Fek1	49	197	718	1194
Fek2	43	95	480	890
Fek3	43	81	365	715
Fek4	47	153	631	1098
Fek5	50	160	598	1090
Fek6	50	169	583	1026
Fek7	52	183	661	1220
Fek8	50	[redacted]		
Fek9	45	177	575	1028
Fek10	43	179	705	1253
Fek11	53	122	380	762
Fek12	45	164	524	950
Fek13	46	177	606	1091
Fek14	42	157	592	1084
Fek15	46	172	664	1230
Fek16	47	168	615	1122
Fek17	46	[redacted]		
Fek18	49	196	660	1155
Fek19	48	134	545	1110
Fek20	48	108	405	1250
Fek21	44	178	605	1066
Fek22	52	123	467	865
Mean	47.18	154.65	568.95	1059.95
SD	3.20	33.17	103.07	154.60

Table S7. Individual gross pathology scores in Groups 1, 2 and 3

**G1 Positive control**	**Score**	**G2 Basal diet + feed additive**	**Score**	**G3 Basal diet + feed additive**	**Score**
ID		ID		ID	
1		21	1	41	
2	2	22	2	42	1
3	1	23	2	43	1
4	1	24	1	44	2
5	0	25	1	45	
6	1	26	1	46	0
7	1	27	2	47	
8	0	28	1	48	1
9	1	29	0	49	1
10	1	30	0	50	
11	4	31	0	51	2
12	1	32	2	52	2
13	4	33	1	53	2
14	0	34	1	54	3
15	1	35		55	1
16	0	36	2	56	
17	3	37	3	57	1
18	1	38	1	58	1
19	0	39		59	1
20	2	40	1	60	0
101	2	102	1	103	2
				Fek23	1
**Mean**	**1.30**		**1.21**		**1.29**

Table S8. Individual gross pathology scores in Groups 4, 5 and 6

G4 Basal diet + feed additive	Score	G5 Basal diet + Amoxicillin	Score	G6 Negative control	Score
**ID**		**ID**		**ID**	
61	1	81	1	Fek1	1
62	0	82	2	Fek2	2
63	1	83	0	Fek3	2
64	1	84	2	Fek4	1
65	1	85	0	Fek5	1
66	0	86	1	Fek6	0
67	2	87	2	Fek7	1
68	0	88	1	Fek8	
69	1	89	2	Fek9	0
70	1	90	1	Fek10	2
71	1	91	0	Fek11	0
72	1	92	1	Fek12	1
73	0	93	2	Fek13	1
74	0	94	2	Fek14	0
75	0	95	1	Fek15	0
76	1	96	2	Fek16	0
77	1	97	2	Fek17	
78	1	98	1	Fek18	0
79	1	99		Fek19	1
80	2	100	1	Fek20	0
104	1	105	2	Fek21	2
		Fek24	2	Fek22	0
**Mean**	**0.81**		**1.33**		**0.75**
